# Ruxolitinib (a JAK2 inhibitor) as an emerging therapy for refractory pruritis in a patient with low-risk polycythemia vera: A case report

**DOI:** 10.1097/MD.0000000000027722

**Published:** 2021-11-05

**Authors:** Abdulrahman F. Al-Mashdali, Waail R. Kashgary, Mohamed A. Yassin

**Affiliations:** a Department of Internal Medicine, Hamad Medical Corporation, Doha, Qatar; b Department of Oncology, Hematology and BMT Section, National Center for Cancer Care and Research, Hamad Medical Corporation, Doha, Qatar.

**Keywords:** Case report, polycythemia vera, pruritis, Ruxolitinib

## Abstract

**Introduction:**

Polycythemia vera (PV) is a Philadelphia-negative myeloproliferative neoplasm (MPN) characterized by the overproduction of red blood cells. The presence of JAK2 mutation is detected in up to 99% of patients with PV. Pruritis is commonly encountered in patients with PV and is considered the most troublesome symptom. Multiple treatment modalities are used for treatment; however, their efficacy is variable. Sometimes, pruritis will not improve even by the use of combined therapies. Recently, Ruxolitinib (a JAK2 inhibitor) has been shown to be very effective, especially in patients with refractory pruritis in the setting of other treatment modalities failure.

**Patient concern:**

We describe a 55-year-old male with history of low risk PV presented with severe itching despite using different therapies, including phlebotomy and hydroxyurea. His laboratory results on presentation were significant for red blood cells (RBC) of 8.2 × 10^6^/uL (normal = 4.5–5.5), hematocrit (Hct) of 52.8% (normal = 40–50%), platelet count of 519 × 10^3^/uL (normal = 150–400), white blood cells count of 12.3 × 10^3^/uL (normal = 4–10), and basophils count of 0.22 × 10^3^/uL (normal < 0.1).

**Diagnosis:**

PV related refractory pruritis.

**Intervention:**

Pruritis improved dramatically after starting Ruxolitinib therapy with an improvement of hematological parameters (both hematocrit and platelet count).

**Conclusion:**

Different treatment modalities have shown to be beneficial in treating PV-related pruritis, but the clinical outcomes are highly variable. This case report aims to shed light on Ruxolitinib as an emerging therapy for the treatment of refractory cases of PV-related pruritis.

## 1 Introduction

Polycythemia vera (PV) is one of the Philadelphia-negative myeloproliferative neoplasm (MPN) characterized by excessive red blood cells production. JAK2 mutation is detected in up to 99% of patients with PV, and exon 14 JAK2V617F mutation is most frequently found. Diagnosis of PV necessitates the presence of a JAK2 mutation, plus increased hemoglobin/hematocrit (>16.5 g/dL or 49% for males and >16 g/dL or 48% for females), based on the 2016 World Health Organization (WHO) revised criteria.^[1,2]^The clinical manifestations of PV are variables, including fatigue, pruritis, features of hyperviscosity, thrombotic events, bleeding complications, and splenomegaly. PV patients with either age above 60 years or a history of thrombosis are classified as having “high-risk” disease, while the absence of both risk factors is considered a “low-risk” disease. This classification into low/high risk is important in PV management.^[2,3]^ Pruritis is reported in 40% of PV patients. Different treatment modalities have been used for pruritis control in PV, including antihistamines, interferon-alpha, phototherapy, phlebotomy, and cytoreductive therapy; however, all of them demonstrated a mixed result.^[4]^ Recently, Ruxolitinib, a JAK2 inhibitor, has been shown to be very effective in the treatment of PV-related pruritis, especially treatment-resistant cases.^[5]^ In this case, we describe a PV patient who presented with severe itching, especially after showering. We tried multiple treatment options for pruritis control but without any considerable benefit. After starting a low dose of Ruxolitinib, we appreciated a dramatic and complete relief of itching without any significant adverse effect.

## 2 Case presentation

A 55-year-old Arabic male with a past medical history of PV presented to the emergency department with severe generalized itching. The patient was diagnosed with PV (positive for exon 14 V617F JAK2 mutation) in 2015, and he has been following in our outpatient clinic since then. There was no history of diabetes mellitus, hypertension, dyslipidemia, thromboembolic event, or smoking. Given his age (<60 years), absence of comorbidities, and no history of a thrombotic event, he was categorized as having a low-risk PV. Accordingly, he was started on regular phlebotomy (hematocrit target below 45%) and Aspirin 100 mg once daily. He underwent a bone marrow biopsy in 2020, which was consistent with the diagnosis of PV. He works as a physician in our hospital.

At the current presentation, the patient complained of severe burning sensation and itching all over the body. He noticed that itching is aggravated by taking a shower, specifically with warm water. He denied any skin redness or rash. He was afebrile and vitally stable. On examination, the patient looked well, and there was no skin discoloration or rash and no organomegaly or lymphadenopathy. His laboratory results on presentation were significant for red blood cells (RBC) of 8.2 × 10^6^/uL (normal = 4.5–5.5), hematocrit (Hct) of 52.8% (normal = 40–50%), platelet count of 519 × 10^3^/uL (normal = 150–400), white blood cells of 12.3 × 10^3^/uL (normal = 4–10), basophils of 0.22 × 10^3^/uL (normal < 0.1), and apart from that all results were within normal range.

The patient was started on hydroxyurea 500 mg twice a day in addition to regular venesection and given a follow-up after one month in the hematology clinic. However, the patient presented again with persistent itching, which significantly affecting his quality of life. Then, a low dose of Ruxolitinib (5 mg twice daily) was started based on the emerging evidence regarding its efficacy in the refractory cases of PV-related itching. At 2 months of follow-up after starting Ruxolitinib, the patient reported a complete resolution of itching, and he was very satisfied with the current therapy. He denied any significant side effects from the new medication. Repeated laboratory investigations revealed a Hct level of 40% (decreased from 52.8%), RBC count of 5.2 × 10^6^/uL (decreased from 8.2), and basophils count of 0.1 × 10^3^/uL (decreased from 0.22).

## 3 Discussion

Pruritis is a well-known defining feature of PV. PV-associated pruritis is commonly induced by water contact, which is recognized as aquagenic pruritis (AP). Sometimes, patients describe PV-associated pruritis as a burning or tingling sensation, often starting after taking a bath. Of note, PV-associated pruritis is not accompanied by skin rash, and the presence of skin lesions should point to other diagnoses. The severity of pruritis in PV is variable and, in some cases, might lead to suicidal ideation. In addition, many patients are considered pruritis as the most upsetting symptom of PV, the same with our patient.^[3,4,6]^

The pathophysiology of PV-associated pruritis is not well-understood. It was found that increased mast cells in the dermis are present in PV patients with pruritis; however, a similar number has been detected in patients without pruritis. Histamine plays a significant role in PV-associated pruritis, and the incidence of pruritis increased by 7-fold in PV patients with raised serum histamine level. Recently, it was found that JAK2V617F mutation-induced cytokine hypersensitivity in the affected hematopoietic cells lineage. Thus, basophils in PV patients are more active and contain more cytoplasmic granules, increasing the risk of pruritis.^[4,7,8]^

Treatment of PV aims to decrease the risk of thromboembolic events, alleviating symptoms (like itching), and lower the risk of transformation to leukemia or myelofibrosis. Nevertheless, management of PV-associated pruritis is primarily empirical. Antihistamines are commonly prescribed for the treatment of this condition, but they are often not effective. Management with phlebotomy reveals mixed outcomes, and it fails to relieve itching in most patients. Improvement of hematological parameters in PV improves pruritis; therefore, some patients with PV-associated pruritis will respond to cytoreductive therapy (like hydroxyurea). However, around 25% of patients will not tolerate hydroxyurea therapy. Although our patient was on both phlebotomy and hydroxyurea therapy, pruritis did not improve at all, and his quality of life was severely affected by that itching.^[2,3,5,6,9]^

Ruxolitinib (JAK1/JAK2 inhibitor) is approved to manage PV patients who are intolerant or resistant to hydroxyurea therapy. Ruxolitinib decreased the expression of cytokines and growth factors required for hematopoiesis by inhibiting JAK1/2- STAT pathways, which explains the improvement in PV-associated pruritis. The RESPONSE study demonstrated that Ruxolitinib is highly effective for the treatment of PV-associated pruritis compared to hydroxyurea. In addition, it showed higher efficacy in controlling hematocrit and reducing splenomegaly.^[5,6,9]^ In our case, the response to Ruxolitinib was dramatic with complete resolution of pruritis. In addition, the hematocrit level was decreased to the target level in PV. On the other hand, our patient tolerated Ruxolitinib therapy and has not reported any side effects over several months of follow-up.

## 4 Conclusion

Pruritis is a common presenting symptom in PV patients and is believed to be the most troublesome manifestation of PV. Different treatment modalities have been shown to be beneficial in treating this condition, but the clinical outcomes are highly variable. Ruxolitinib is an emerging therapy for refractory cases of PV-associated pruritis. It is more effective than hydroxyurea in pruritis control, and it is well tolerated with a better safety profile. Hence, we recommend it as a first-line treatment of refractory pruritis in PV patients.

## Acknowledgments

We would like to acknowledge the Qatar National Library (QNL) for open access publication funding of this article.

## Author contributions

**Conceptualization:** Abdulrahman Fadhl Al-Mashdali, Mohamed A. Yassin.

**Data curation:** Abdulrahman Fadhl Al-Mashdali, Mohamed A. Yassin, Waail Rozi Kashgary.

**Writing – original draft:** Abdulrahman Fadhl Al-Mashdali, Mohamed A. Yassin, Waail Rozi Kashgary.

**Writing – review & editing:** Abdulrahman Fadhl Al-Mashdali, Mohamed A. Yassin.
